# Vascular endothelial cellular mechanics under hyperglycemia and its role in tissue regeneration

**DOI:** 10.1093/rb/rbae004

**Published:** 2024-01-25

**Authors:** Kui Wang, Yongmei Ge, Yongshuai Yang, Zhenjian Li, Jiayi Liu, Yizebang Xue, Yuanjun Zhang, Xiangchao Pang, A H W Ngan, Bin Tang

**Affiliations:** Department of Biomedical Engineering, Southern University of Science and Technology, Shenzhen 518055, China; Department of Mechanical Engineering, University of Hong Kong, Hong Kong 999077, China; Department of Biomedical Engineering, Southern University of Science and Technology, Shenzhen 518055, China; Department of Biomedical Engineering, Southern University of Science and Technology, Shenzhen 518055, China; Department of Biomedical Engineering, Southern University of Science and Technology, Shenzhen 518055, China; Department of Biomedical Engineering, Southern University of Science and Technology, Shenzhen 518055, China; Department of Biomedical Engineering, Southern University of Science and Technology, Shenzhen 518055, China; Department of Biomedical Engineering, Southern University of Science and Technology, Shenzhen 518055, China; Guangdong Provincial Key Laboratory of Cell Microenvironment and Disease Research, Shenzhen Key Laboratory of Cell Microenvironment, Shenzhen 518055, China; College of Materials Science and Engineering, Central South University of Forestry and Technology, Changsha 410004, China; Department of Mechanical Engineering, University of Hong Kong, Hong Kong 999077, China; Department of Biomedical Engineering, Southern University of Science and Technology, Shenzhen 518055, China; Guangdong Provincial Key Laboratory of Cell Microenvironment and Disease Research, Shenzhen Key Laboratory of Cell Microenvironment, Shenzhen 518055, China

**Keywords:** endothelial cell, hyperglycemia, stiffness, migration, Cdc42

## Abstract

Diabetes is one of the most prevalent diseases worldwide. The tissue regeneration of diabetes patients is known to be rather tricky as the result of vascular dysfunction, and this leads to various clinical complications including diabetic foot ulcers. The vascular endothelial cells, which compactly line the inner surface of blood vessels, are responsible for the growth and maintenance of blood vessels and play an essential role in tissue regeneration. Although the mechanical properties of cells are generally known to be regulated by physiological/pathological conditions, few studies have been performed to investigate vascular endothelial cellular mechanics under hyperglycemia and the biological functions related to tissue regeneration. In this study, we conduct a systematic investigation of this issue. The results suggested that the stiffness of human umbilical vein endothelial cells (HUVECs) can be significantly regulated by the glucose concentration, subsequently, leading to significant alterations in cell migration and proliferation capabilities that are closely related to tissue regeneration. The rearrangement of the cytoskeleton induced by hyperglycemia through Cdc42 was found to be one of the pathways for the alteration of the cell stiffness and the subsequent cell dysfunctions. Therefore, we suggested that the inhibition of Cdc42 might be a promising strategy to facilitate various tissue regeneration for diabetes patients.

## Graphical Abstract

**Figure:**
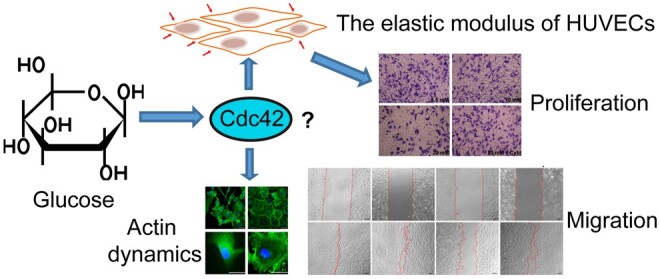

## Introduction

Diabetes is a prevalent health issue globally, characterized by chronic hyperglycemia and metabolic syndrome. Moreover, the tissue regeneration of diabetes patients is largely delayed and leads to complications such as diabetic foot ulcers [1]. Although it is generally believed that the lack of tissue regeneration capabilities in diabetes should be due to endothelial dysfunction, the exact mechanism is still ambiguous and needs to be further explored [2].

Angiogenesis, the growth of new blood vessels or neovascularization to nutrify damaged tissues, is critical to various tissue regeneration [1]. Endothelial cells, especially their motility and proliferation ability, play critical roles in angiogenesis and the tissue regeneration process. The initiation of endothelial cell migration is essential to restore the normal integrity of the lining of vascular endothelial cells and the repair mechanism of the tissue regeneration process [3]. However, endothelial dysfunction is the earliest and most fundamental pathological change in diabetes and leads to the reduction of tissue regeneration capabilities. Therefore, it is imperative to clarify the detailed mechanism for the hyperglycemia environment causes endothelial dysfunction, in order to find possible solutions to facilitate the regeneration process.

Elastic modulus, or sometimes in terms of stiffness, is a vital mechanical property of cells that is attracting increasing attention from researchers, especially for its correlation with cellular motility. Cross *et al*. showed that metastatic cancer cells, which exhibit more vigorous motility than their non-cancerous counterparts, are also much softer [4]. In addition, other non-cancer cells, including mesenchymal stem cells [5], tenocytes [6], embryonic fibroblasts [7] and epithelial cells [8], were also found to have cellular stiffness closely related to their migration capability. However, the most common criteria for determining whether endothelial cells are dysfunctional by hyperglycemia are viability damage, metabolic disorder and inflammatory response [9]. So far, there has been no attempt to consider physical properties such as stiffness and plasticity as indicators of dysfunction of endothelial cells, despite their obvious association with cellular motility and wound healing.

Several factors have been reported *in vitro* to increase the stiffness of endothelial cells, such as exposure to shear stress [10], pro-inflammatory cytokine tumor necrosis factor-α (TNF-α) [11] and oxidized low-density lipoprotein (oxLDL) [12, 13]. Gap-junction-mediated endothelial cell–cell interaction and TNF-α-mediated endothelial cellular stiffening are known to be modulated by cytoskeletal rearrangement and focal adhesion formation [14]. OxLDL-induced stiffening of endothelial cells is mediated by the RhoA/Rho kinase pathway [12]. *Escherichia coli* cytotoxic necrotizing factor, a toxin which, in addition to Rho, activates the GTPases Rac1 and Cdc42, is also reported to induce a dramatic stiffening effect, suggesting that the stiffening may be mediated through the Rac1- or Cdc42-dependent pathway [15]. Cytoskeletal rearrangement also plays a significant role in the regulation of cellular stiffness [16], and both the actomyosin cytoskeleton and filamentous actin (F-actin) are important determinants of cellular stiffness [17, 18]. However, it is still ambiguous whether the hyperglycemia condition can regulate the stiffness of human umbilical vein endothelial cells (HUVECs) and its exact role in the tissue regeneration process.

It has been reported that the GTPase family members, mainly RhoA, Rac1 and Cdc42, have specific effects on the actin filament system and, thereby, on the morphology and migration of cells [19]. These three genes, *RhoA*, *Rac1* and *cdc42*, can evoke cytoskeleton organization restructuring in diverse manners and act differently in the activation of actin polymerization, with RhoA controlling the generation of stress fibers and focal adhesions [20], Rac1 promoting the formation of lamellipodia [21] and Cdc42 supporting the formation of filopodia [22]. Subsequently, all of these three proteins influence the mobility of cells significantly. They can regulate the G-actin to polymerize into F-actin to generate a specific structure through activating proteins of the Wiskott Aldrich syndrome protein (WASP) family, which activates the actin-related protein Arp2/3 complex that initiates the nucleation of the new actin filaments to form branched actin filament networks [23]. The GTPase-mediated cytoskeleton rearrangement can also affect cell stiffness. Sharma *et al*. observed that activating the Rho isoforms RhoA, Rac1 and Cdc42 caused the cytoskeleton to reorganize into long F-actin filaments and be more spread out on the membrane and so result in an increased cell stiffness [24].

To understand the effect of hyperglycemia on HUVECs mechanics and the migration capabilities subsequently, this study investigates the elastic modulus of HUVECs in different glucose concentrations by atomic force microscope (AFM) nanoindentation via a rate-jump method [25, 26]. Then the migration ability is evaluated by wound healing and transwell method, respectively. The results demonstrate that the Cdc42 plays an important role in the process of hyperglycemia-induced HUVECs dysfunctions by changing the elastic modulus of HUVECs. This study reveals the fact that HUVECs are stiffened and then lose their migration capabilities due to hyperglycemia condition, which should provide new inspirations in potential strategies for better-treating wound healing and facilitating the tissue regeneration of diabetes patients.

## Materials and methods

### Cell culture

Human umbilical vein endothelial cells (HUVECs, E680518-0001, Sangon Biotech, Shanghai, China) were maintained in Dulbecco modified Eagle medium (DMEM, Hyclone, USA) without glucose and supplemented with 10% FBS (Hyclone, USA), 100 U/ml penicillin and 100 µg/ml streptomycin (Beyotime, China) at 37°C in 5% CO_2_. To mimic the glucose condition within the diabetic range, the HUVECs were treated with media containing different glucose concentrations (5.5, 10 or 20 mM). To exclude the possible influence of osmolarity on cellular biochemical and biophysical behavior, the mannitol (14.5 and 10 mM) was added to the medium with 5.5 and 10 mM glucose.

### Single-cell force spectroscopy

**Figure 1.**
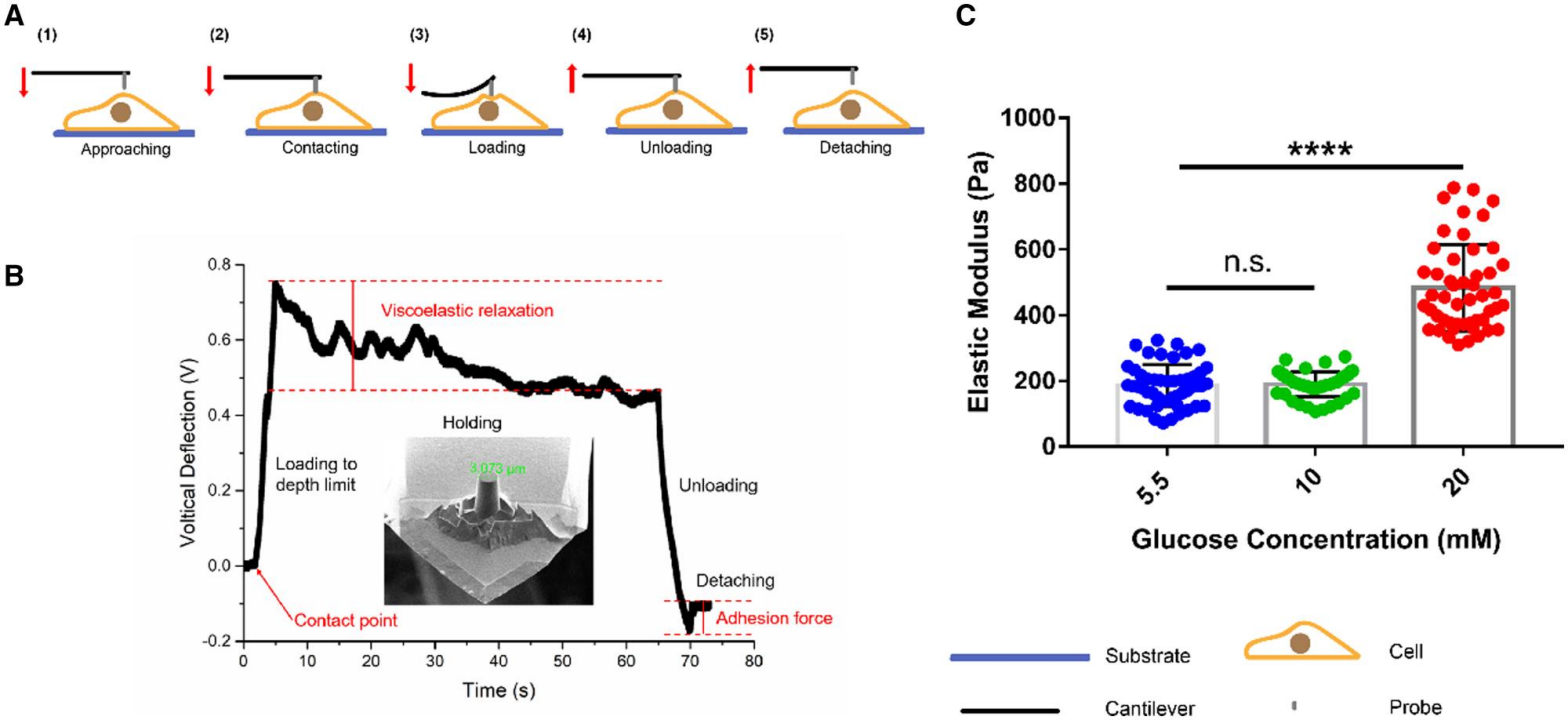
The elastic modulus of HUVECs in different glucose concentrations. (**A**) Schematic diagram of the measurement procedure of the elastic modulus of HUVECs by AFM; (**B**) the representative curve of force spectroscopy of HUVECs. The inserted is the scanning electron microscope (SEM) picture of the used probe in this study (*d* = 3.022 µm); (**C**) The elastic modulus of HUVECs in different glucose concentrations. *N* = 50. ****P <* *0.0001.

The force measurement on HUVECs was carried out at 37°C in PBS in an atomic force microscope (JPK Nano Wizzard II, version 5.0.135, Bruker, USA) equipped with a heater to stabilize the temperature. The silicon nitride tips (CSG01, NT-MDT) were modified by focused ion beam milling (FIB, Helios NanoLab 650/600i, FEI company, USA) to create a flat-ended cylinder shape of 3 µm diameter, as shown in the insert of Figure 1B. The sensitivity of the system and the spring constant of the cantilever were calibrated by performing indentation tests on a clean silica surface in water and the thermal noise method, respectively. The instrumentation details of the AFM measurement have been described elsewhere [27]. During the AFM measurements, in brief, the AFM tip was made to indent into the cell specimen after first making contact with it, by driving the piezo which moved the clamped end of the AFM cantilever at a speed of 0.1 µm/s for 10 s, followed by holding the piezo height for another 60 s. The force spectroscopy steps and a typical force-depth curve are shown in Figure 1A and B, respectively. After holding, the tip was retracted to its original position at a piezo speed of 0.1 µm/s. The piezo height and cantilever deflection signals were simultaneously collected during the entire indentation measurement.

To obtain the elastic modulus of HUVECs, the rate-jump method was employed to eliminate viscoelastic effects on the measured elastic modulus values [26]. In this method, the elastic modulus of the samples was calculated by the following equation [25]:

$$\Delta \dot{F}=2a(\frac{E}{1-\nu^{2}})\Delta \dot{h}$$

where $\dot{h}$ and $\dot{F}$ are rates of the indentation depth and indentation force, respectively; E is the sample’s Elastic modulus; ν is the sample’s Poisson ratio which is assumed to be 0.5 in this research; a is the radius of the flat-ended cylindrical tip (1.5 µm in this research); $\Delta \dot{F}$ and $\Delta \dot{h}$ are the jumps in $\dot{h}$ and $\dot{F}$ between the end of the holding segment and the beginning of the retracted segment, respectively. In this study, the indentation force F was evaluated as F = k × h, where k is the spring constant of the AFM cantilever, and h is the deflection of the cantilever.

### Immunofluorescence staining

A laser scanning confocal microscope (LSCM, Leica, Germany) was employed to visualize the cytoskeleton protein and nucleus. After the HUVECs were cultured in different glucose concentrations for 24 h, the cells were fixed with 4% formaldehyde (Beyitime, China) for 10 min. The F-actin was stained with FITC-conjugated phalloidin (1:200) (Sigma, USA), and the nuclei were stained with 1 µg/ml 40, 60-diamidino-2-phenylindole (DAPI, Sigma, USA). The Image J software (Version: 1.52V) was used to process the LSCM images.

### G-actin/F-actin ratio assay

In order to quantify the F-actin ratio incorporated into the cytoskeleton and G-actin found in the cytosol, a G-actin/F-actin *in vitro* assay kit was used (Cytoskeleton Inc, Denver, CO, USA). In brief, the HUVECs were lysed by an F-actin stabilization buffer and then centrifuged to separate the F-actin from the G-actin pool at 100 000 × *g* at 37°C for 1 h. The F-actin was resuspended in an F-actin depolymerization buffer, and then the F-actin and the G-actin were separated by sodium dodecyl sulfate-polyacrylamide gel electrophoresis. Western blotting (WB) was used to quantitatively analyze the separated proteins. The ratio of F-/G-actin was calculated by the ImageJ software.

### Reverse transcription-polymerase chain reaction

Total RNA was isolated by using RNAiso Plus (TaKaRa) and then synthesized into cDNA by using the cDNA Synthesis Kit (Beyotime, China). Real-time PCR was performed with a SYBR Green Real-time PCR Master Mix kit (Beyotime, China). Real-time PCR was performed on an Applied Biosystems, StepOnePlus Instrument (USA) with the following cycling program: 95°C for 5 min pre-degeneration, followed by 40 cycles of amplification at 95°C for 15 min and 60°C for 60 s, then 95°C for 15 s. The gene expression level was normalized by GADPH, and ΔΔCT was calculated by referring to the control group. The primers used are listed in Table 1.

**Table 1.** The sequence of the primers for reverse transcription-polymerase chain reaction

Gene name	Forward primer (5′–3′)	Reverse primer (5′–3′)
*cdc42*	CCATCGGAATATGTACCGACTG	CTCAGCGGTCGTAATCTGTCA
*WASP*	CCCCAAATGGTCCTAATCTACCC	TGGAAATTGCTTGGTGTTCCTAT
*RhoA*	GGAAAGCAGGTAGAGTTGGCT	GGCTGTCGATGGAAAAACACAT
*Rac1*	ATGTCCGTGCAAAGTGGTATC	CTCGGATCGCTTCGTCAAACA
*GAPDH*	AGGGCTGCTTTTAACTCTGGTAAA	GAATTTGCCATGGGTGGAAT

### Small interfering (si)RNA transfection

To silence the expression of *cdc42* gene, a set of on-target human siRNAs was synthesized (Ribobio, China). The sequence of the siRNAs was Cdc42-siRNA (GCAAGAGGATTATGACAGA) and a scrambled control-siRNA (AGCAGATGATAGCCATGAAGT). Briefly, HUVECs were seeded the day before the transfection to reach 70–80% confluence and were then transfected with 100 pM of siRNA using the LipoRNAi^TM^ reagent (Biyotime, China) following the suggested protocol. After transfection for 12 h, the culture medium was replaced by a fresh complete medium. Cells were kept under culture conditions up until 48 h post-transfection and then processed for further experiments. siRNA efficiencies were measured by qPCR and WB.

### Cell viability assay

The MTT assay was employed to test the viability of HUVECs in different glucose concentrations. Briefly, 5 × 10^3^ HUVECs were seeded in 96-well plates in standard medium for attachment in 4 h and then cultured in 5.5, 10 or 20 mM glucose concentration media for 1, 3 and 5 days. The 3‐(4,5‐dimethylthiazol‐2‐yl)‐2,5‐diphenyltetrazolium bromide solution (5 mg/ml) was added to each well and incubated for 4 h at 37°C. Then the formazan was dissolved in dimethylsulfoxide (Sangon Biotech, China), and the absorbance of each well was measured by an iMark Microplate Reader (Bio-rad, USA) at a wavelength of 570 nm.

### Transwell migration assay

To reveal the effects of hyperglycemia on the migration behavior of HUVECs, a transwell system (Corning, USA) was used. In brief, the HUVECs were treated with different glucose concentrations (5.5 or 20 mM) for 24 h and then starved for 6 h. The high-glucose (HG) condition (20 mM) was also added with or without 1 µM ML141 (TSBioChem, China) which is a Cdc42 inhibitor, siRNA of Cdc42 and scrambled negative control RNA, respectively. To dismiss the osmotic influence, the normal glucose condition (5.5 mM) was also added with 19.5 mM mannose. A total of 200 µl DMEM containing 1 x 10^5^ cells without FBS was transferred into the upper chambers (8 µm pores), while the bottom chamber was pooled with 600 μl of cell-free DMEM containing 10% FBS. The cells were allowed to migrate for 24 h. Then, the non-migrated cells were removed by cotton swabs, and the migrated cells on the bottom were fixed with 4% paraformaldehyde and stained with 1% crystal violet. Then the migrated cells were counted in microscopy in 8 areas.

### Wound healing assay

HUVECs were seeded at density 1x10^5^ cells/mL and cultured in 60 mm tissue culture dishes to 80–90% confluence and then were serum-starved for 24 h. Then, a wound was made in the confluent monolayer by a sterile yellow tip. Following washing twice with PBS, the cells were incubated in DMEM with normal glucose (5.5 mM), and 20 mM as a high concentration. The high-concentration groups were treated by 1 µM ML141, siRNA of Cdc42 and scrambled negative control RNA, respectively. Images of the same regions were captured at 0 and 24 h with a light microscope.

### Statistical analysis

The statistical significance of the differences between the two groups was assessed by unpaired T-test. One-way ANOVA was used to evaluate the differences between the multi-group single-variable experiments, and two-way ANOVA was used to evaluate the differences between the multi-independent experiments. They were expressed in the format of mean ± standard error of the mean. Data were analyzed using the GraphPad Prism 8 software for Windows, and differences were considered statistically significant when the *P* values were less than 0.05.

## Results

### Hyperglycemia increases the elastic modulus of HUVECs *in vitro*

To figure out the effective concentration of glucose on endothelial cell stiffness, HUVECs were cultured in 5.5, 10 and 20 mM glucose for 24 h and stiffness was then measured. There were five stages of a cell measured by the atomic force microscope and the direction of probe displacement through cartoon drawings, including approaching, contacting, loading, unloading and detaching (Figure 1A). It showed the microstructure of the cut cylindrical probe and a typical curve of cell mechanics measurement in Figure 1B. Key points in the measurement are marked, including the contact point, the loading mechanics process, the viscoelastic effect of cells in holding, the process of unloading force, the unloading point and the adhesion. As shown in Figure 1C, the elastic modulus of HUVECs increased with the increased concentration of glucose and showed the highest value of 550 Pa at 20 mM.

### Hyperglycemia facilitates the stress fiber assembly of HUVECs

**Figure 2.**
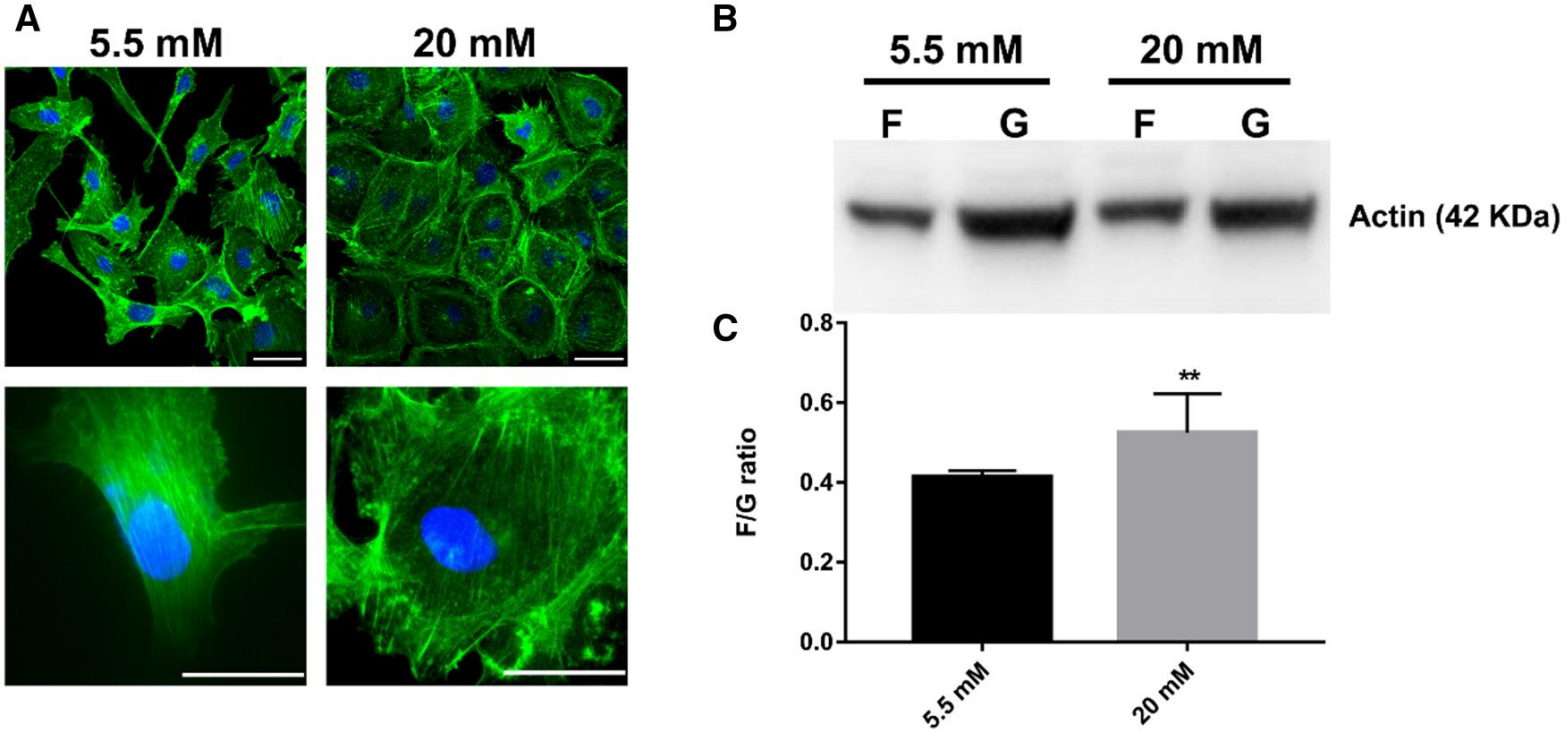
Glucose concentration effects on actin dynamics. (**A**) Representative images of F-actin cytoskeleton of HUVECs. After 24 h of incubation with different glucose conditions, cells were fixed and stained. Green represents F-actin labeling with FITC-conjugated phalloidin, and blue represents the nucleus stained with DAPI. The upper two images were visualized in 40×, and the lower two images were visualized in 100×, bar: 50 µm; (**B**) representative Western blot of F-/G-actin in HUVECs in 5 mM and 20 mM during 24 h; (**C**) quantification of F-/G-actin ratio in HUVECs in NG (5 mM) or HG (20 mM) during 24 h. Data are expressed as means ± SD of at least three experimental values. **P <* *0.01.

It should be noted that the maximum indentation depth was around 2 µm during the nanoindentation, so the measured mechanical response should be mainly due to the deformation of the cytoskeleton. Through fluorescence imaging of actin, the number of stress fibers was found to be higher (about 1.3-fold) in the hyperglycemia condition than in the normal state (Figure 2A and Supplementary Figure S1). In normal condition, the cells were in an anisotropic spindle shape with a small spread area comprising dense stress fibers stretching across the leading edge to the rear edge. However, in the HG condition, the cells adopted an isotropic circular shape with a large spread area and a well-formed actin cortex located at the outskirt of the cells. The stress fibers and actin cortex were formed by F-actin, which was polymerized through G-actin. The F-/G-actin ratio for HUVECs was also found to be increased in the hyperglycemia state (Figure 2B and C).

### Hyperglycemia inhibits the migration and proliferation of HUVECs

**Figure 3.**
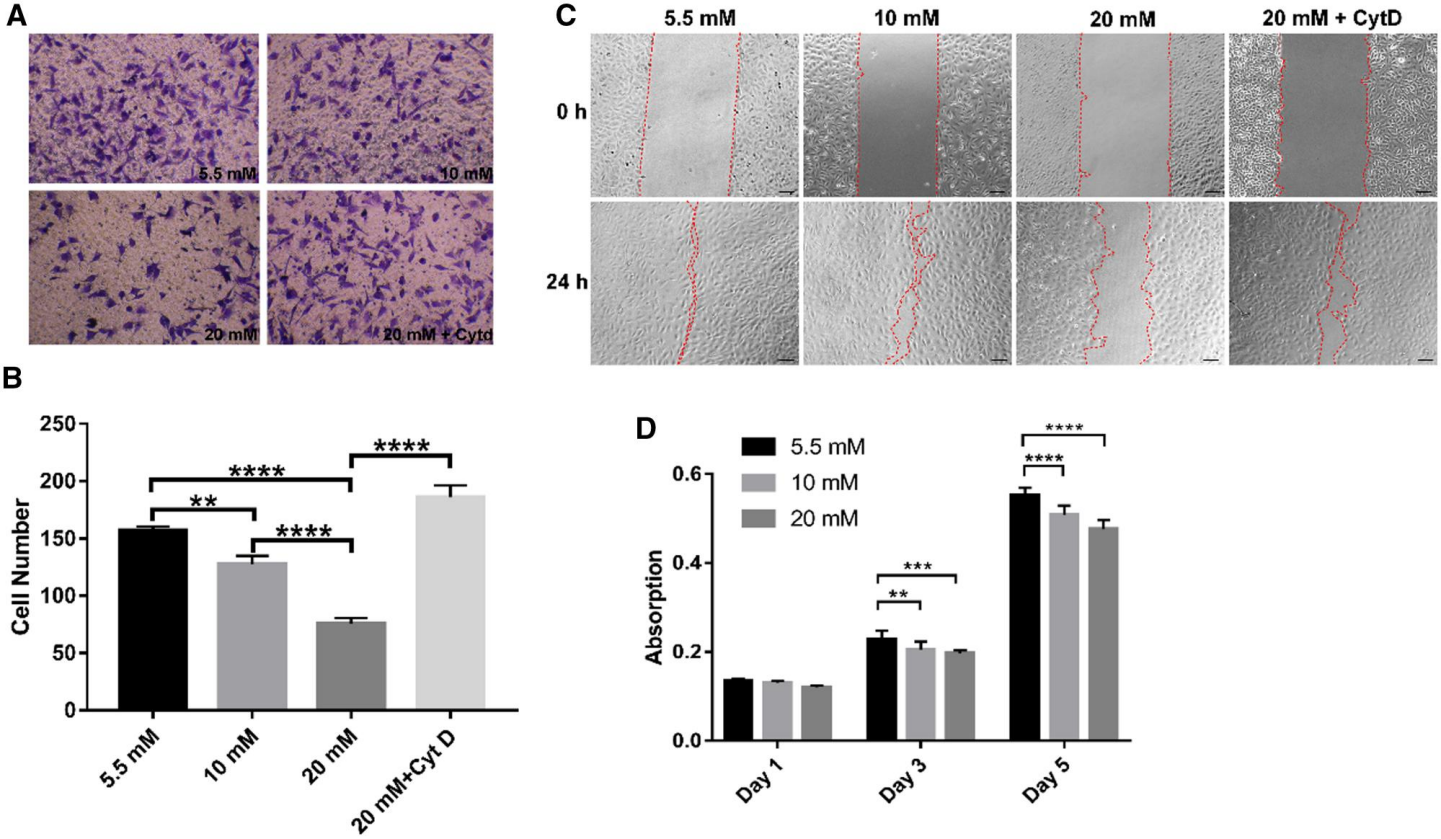
HG Inhibited the migration and proliferation of HUVECs. (**A**) The typical transwell cell picture stained by 1% crystal violet in 5.5 mM, 10 mM, 20 mM, or 20 mM + CytD condition, respectively; (**B**) an average number of transwell cells of three independent experiments; (**C**) the typical wound healing image of HUVECs in 5.5mM, 10 mM, 20 mM or 20 mM + CtyD condition, respectively; (**D**) cell growth rates of HUVECs in 5.5, 10 and 20 mM glucose concentration in 1, 3 and 5 days by MTT assay. Bars, SD. **P <* *0.01, ***P <* *0.001, ****P <* *0.0001.

To examine the migration capabilities of HUVECs, a transwell migration and a wound healing assay were used to evaluate cell migration in 3D and 2D in different glucose concentrations. HUVECs exhibited reduced migration capabilities in 20 mM compared with 5.5 mM both in 3D and 2D (Figure 3A–C). To validate the relationship between cytoskeleton stiffness and cell migration capabilities, cytochalasin D (CytD) was used to block the stress fiber assembly to reduce the stiffness of HUVECs under hyperglycemia condition [28]. We observed that CytD treatment successfully restored the migration capabilities of HUVECs while concurrently reducing their elastic modulus by approximately 60% in comparison with the control group (Figure 3A–C and Supplementary Figure S2). From the wound closure rates, the effect of CytD was similar to that of the 5.5 mM group (Supplementary Figure S3). In addition, the wound closure rate of the 20 mM glucose group was only about 70% of that of the 5.5 mM group (Supplementary Figure S3). Then, we also explored the proliferation of HUVECs in different glucose concentrations by MTT. The results showed that the HUVECs’ proliferation was significantly inhibited under hyperglycemia conditions (Figure 3D).

### Cdc42 is overexpressed in HUVECs in hyperglycemia

**Figure 4.**
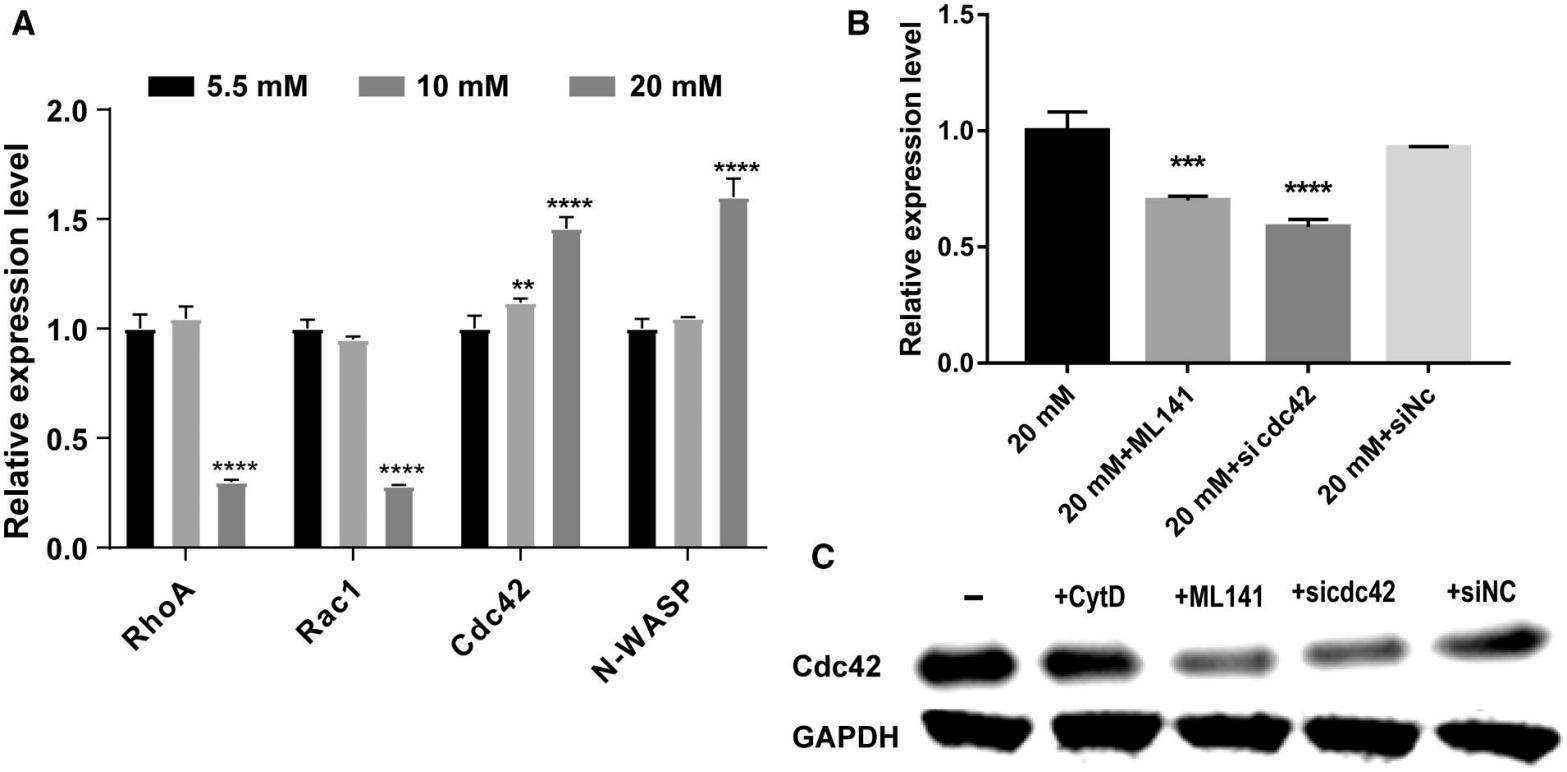
Cdc42 was overexpressed in HG. (**A**) qRT-PCR for *RhoA*, *Rac1*, *cdc42* and *N-WASP* was performed with cDNA from HUVECs in different glucose concentrations (5.5, 10 and 20 mM). Data were analyzed by the ΔΔCt method and were depicted as fold-change expression relative to control cells (set to 1, 5.5 mM) normalized to the housekeeping gene GAPDH (*n* = 3); (**B**) qRT-PCR for detecting *cdc42* gene expression treated by ML141 (1 µM), si*cdc42* and siNC in HG; (**C**) expression of *cdc42* was respectively confirmed by Western blotting in CytD (1 µM), ML141, si*cdc42* and siNC in HG. **P <* *0.01, ***P <* *0.001, ****P <* *0.0001.

The GTPase family, including RhoA, Rac1 and Cdc42, is known to play an important role in the rearrangement and polymerization of the cytoskeleton. Therefore, the expression of *cdc42* and the downstream genes *WASP*, *RhoA* and *Rac1* was compared in NG (5.5 mM) and HG (20 mM). HUVECs with HG showed a high expression of *cdc42* and WASP and a low expression of *Rac1* and *RhoA*, compared with NG (Figure 4A). To further study the role of the *cdc42* gene, the specific inhibitor ML141 or RNA interference (RNAi) was used to inhibit the expression of *cdc42* in HG. qPCR and WB revealed a reduction of *cdc42* in gene and protein levels both by inhibitor ML141 and RNAi in HG (Figure 4B and C). The expression levels of Cdc42 in the ML141 and sicdc42 groups decreased by approximately 26% and 19% compared to the control group, respectively (Supplementary Figure S4). We also found that the CytD did not influence the Cdc42 protein expression (Figure 4c and Supplementary Figure S4).

### Inhibited Cdc42 decreases the elastic modulus of HUVECs through interfered stress fiber assembly in hyperglycemia

**Figure 5.**
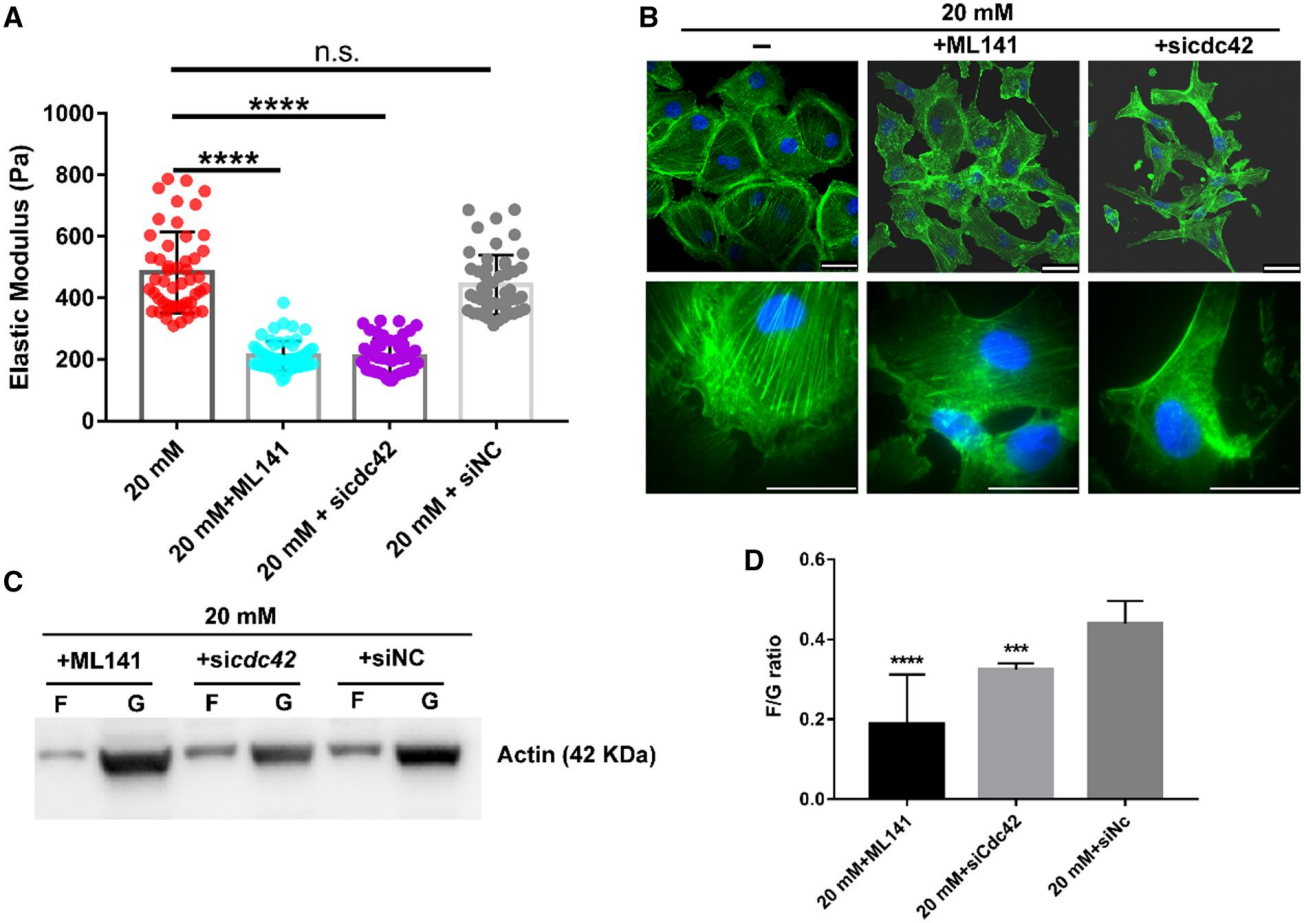
Inhibiting Cdc42 decreases the elastic modulus of HUVECs through interfered stress fiber assembly in HG. (**A**) Elastic modulus of HUVECs treated by Cdc42 inhibitor or RNAi in HG, *N* = 50; (**B**) representative images of F-actin cytoskeleton of HUVECs. After 24 h of incubation with ML141 (1 µM) or si*cdc42* in HG, cells were fixed and stained. Green represents F-actin labeling with FITC-conjugated phalloidin, and blue represents the nucleus stained with DAPI. The upper three images are visualized in 40×, and the lower three images in 100×, bar 50 µm; (**C**) representative Western blot of F-/G-actin of HUVECs treated by ML141 (1 µM) or si*cdc42* or siNC in HG; (**D**) quantification of F-/G-actin ratio of HUVECs treated by ML141 (1 µM) or si*cdc42* or siNC in HG. Data in means ± SD of at least three experimental values. ***P <* *0.001, ****P <* *0.0001.

To further explore the relationship between the *cdc42* gene and the elastic modulus of HUVECs, the elastic modulus of HUVECs treated by si*cdc42* or ML141 to downregulate the expression of *cdc42* was measured using AFM in the HG condition. In Figure 5A, the elastic modulus of HUVECs in the HG condition was decreased to 200 and 210 Pa after inhibition of *cdc42* expression by inhibitor ML141 and si*cdc42*, respectively. On the other hand, the elastic modulus of HUVECs treated only with siNC in HG was almost equal to the untreated group in the same HG condition. From the F-actin fluorescence images, the stress fibers assembled by F-actin decreased severely after inhibition of *cdc42* expression with inhibitor ML141 or si*cdc42*, compared with the untreated group in the same HG condition (Figure 5B). The cells also transformed from an isotropic circular shape to an anisotropic spindle shape with a reduced spread area after inhibition of *cdc42* expression through inhibitor ML141 and si*cdc42* (Figure 5B). The F-/G-actin ratio also decreased with *cdc42* expression inhibition by ML141 and si*cdc42* (Figure 5C and 5D).

### Inhibiting cdc42 expression restores the migration of HUVECs in hyperglycemia

**Figure 6.**
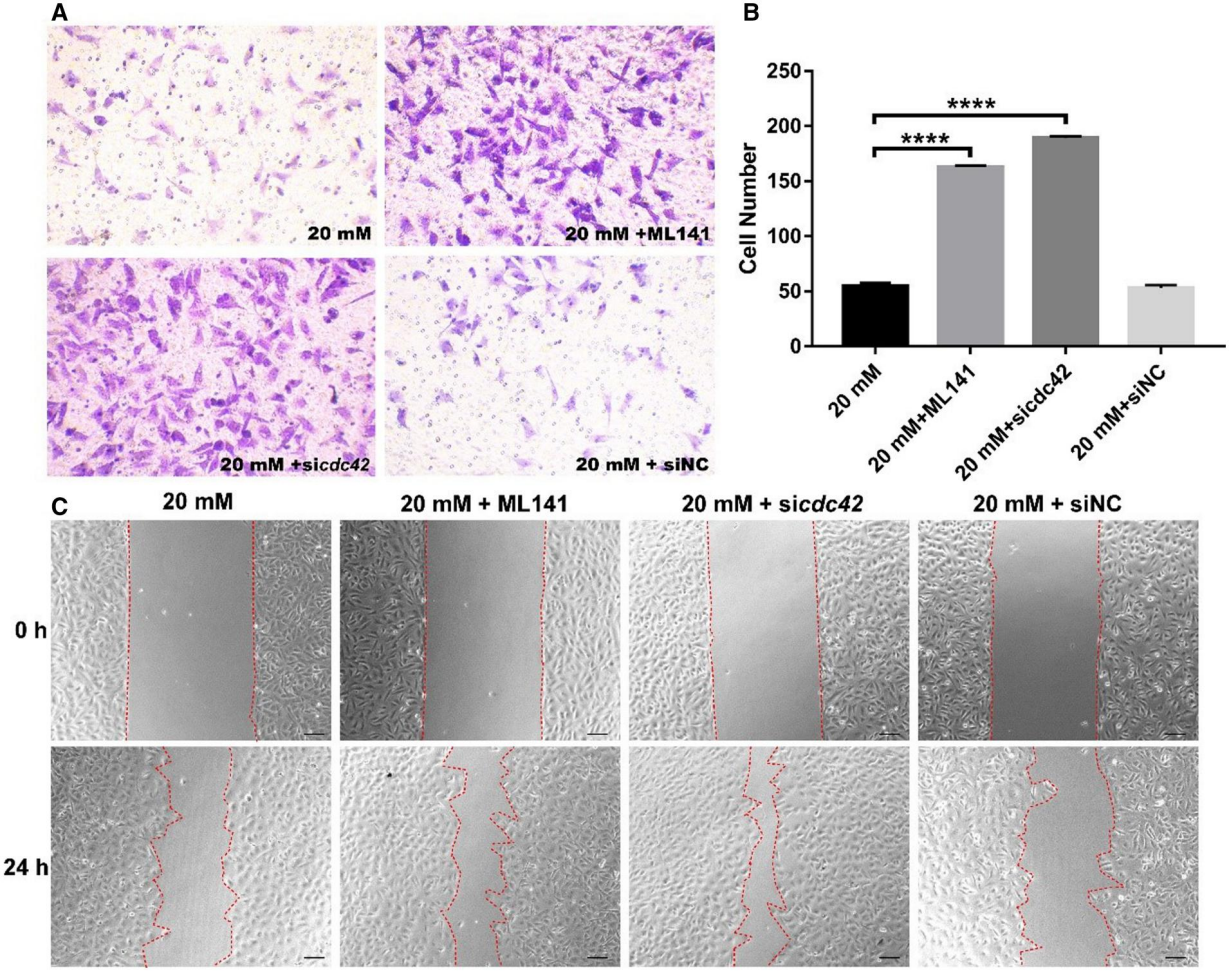
Inhibiting *cdc42* expression restores the migration of HUVECs in HG. (**A**) Typical images of transwell cells stained by 1% crystal violet in 20 mM, 20 mM + ML141, 20 mM + si*cdc42*, or 20 mM + siNC condition, respectively; (**B**) average number of transwell cells of three independent experiments; (**C**) typical wound healing images of HUVECs in20 mM, 20 mM + ML141, 20 mM + si*cdc42*, or 20 mM + siNC condition, respectively. ****P <* *0.0001.

To further inspect the relationship between the *cdc42* gene and HUVECs function, we employed transwell and wound healing methods to detect the migration ability of HUVECs in 3D and 2D after inhibiting the *cdc42* gene through inhibitor ML141 or si*cdc42* in HG condition. The transwell experiment in Figure 6A and B showed that the migration ability of HUVECs was immensely resumed when *cdc42* expression was inhibited by inhibitor ML141 and si*cdc42* in 3D. From the wound healing results, treating HUVECs with ML141 and sicdc42 to inhibit Cdc42 expression resulted in a reduction in the size of the healed gap (Figure 6C). Under this high-glucose condition, their wound closure rates were 1.2-fold and 1.3-fold higher compared to the 20 mM control group or the siNC treatment group (Supplementary Figure S3). These results suggest that inhibiting *cdc42* expression would promote the migration of HUVECs in HG condition in 2D.

## Discussion

In diabetic patients, endothelial dysfunction is a common occurrence that causes the delayed tissue regeneration and then precedes the development of diabetes-related vascular complications such as diabetic foot ulcers and strokes. Although recent studies have highlighted the significant role of biomechanical properties in endothelial function, the impact of hyperglycemia on the mechanical properties and function of HUVECs remains unclear. Our findings suggested that under HG conditions, HUVECs become notably stiffer due to the promotion of stress fiber assembly and a decrease in migration ability. Furthermore, the increased elastic modulus is closely related to regulatory changes in the transcription and expression of *cdc42* and its downstream gene *N-WASP*, both of which respond to stress fiber assembly. We also prove that the stiffer HUVECs exhibit deceased migration capabilities as compared with normal ones. Therefore, our study might give some hints to the detailed relationship between hyperglycemia and tissue regeneration: under HG conditions, the stress fiber assembly of HUVECs was significantly changed and led to the stiffen of HUVECs via regulating the *cdc42* expression. The increase in cell stiffness will result in the loss of its migration and proliferation capabilities, and so contribute notably to endothelial dysfunction, and subsequently, the loss of capabilities in tissue regeneration. In other words, inhibiting the *cdc42* expression, and so soften the HUVECs, might be a possible solution to promote tissue regeneration for diabetic patients.

Collectively, these findings provide evidence that the hyperglycemia condition leads to increased stiffness in HUVECs, resulting in impaired migration and proliferation functions. This effect is mediated by the upregulation of Cdc42, which further promotes stress fiber assembly. Several studies have reported that hyperglycemia would lead to the dysfunction of endothelial cells, embracing acceleration of endothelial cell senescence, dysregulation of nitric oxide synthase and promotion of local inflammation [9]. The present results indicate that the stiffness of endothelial cells detected by AFM would be impacted by hyperglycemia too. The elastic modulus of endothelial cells measured in previous research falls in a very wide distribution range, from 385.5 ± 131.67 Pa to 3.0 ± 1.6 kPa [29, 30]. One reason for such large variation is cellular viscoelasticity which is known to severely affect the accuracy of elastic modulus measurement [25]. In this work, the measurement should be reliable because the rate-jump method used is proven to be valid for removing viscoelastic effects in elastic modulus measurement in soft matters, including gels and cells [26, 31, 32]. Another reason for the large variation in the literature data is the use of sharp AFM tips of nanometric sizes, which may indent into the gaps or the cytoskeleton protein of cells. In this work, a cylindrical AFM tip with a larger flat end of 3 µm diameter was used, which would ensure that the cytoskeleton was probed every time.

The actin cytoskeleton provides the basic infrastructure for maintaining the optimal mechanical behavior of the cell and is the primary driver of cell migration. Therefore, the mechanical properties of cells are closely related to their mobility through the linkage of the actin cytoskeleton [33–36]. F-actin, formed by the polymerization of G-actin, is the basic structural unit that forms the actin cytoskeleton of cells. Therefore, the density of local F-actin is closely related to the stiffness of cells [37]. In the present work, we also observed that the F-/G-actin ratio of HUVECs increased in the hyperglycemia condition, which means that the hyperglycemia induced polymerization of G-actin into F-actin, leading to cell stiffness increment as a result of the increased F-actin density. Cell plasticity and morphology are known to reflect the migratory behavior of cells [38, 39], which in turn is governed by cell mechanics that are determined by the actin cytoskeleton.

It is well known that active Cdc42, one of the major Rho GTPase family members, acts as molecular switches to regulate cytoskeletal reorganization, format long, parallel and tight bundles to construct the filopodia, and then govern cell migration [40]. However, some researchers have drawn contrary conclusions on the role of Cdc42 in cellular migration. Liu *et al*. observed that SCAP-Exo improved cytoskeletal reorganization and contributed to the migration of endothelial cells via Cdc42 signaling [41]. Li *et al*. found Eva1a upregulated Cdc42 expression level and then promoted the HAECs migration [42]. On the contrary, Shen *et al*. demonstrated that elevated activation of Cdc42 resulted in a larger surface area and reduced migration in NR1-sh podocytes [43]. Liu *et al*. reported that DAPT inhibited the migration of breast cancer cells by activating Cdc42 [44]. Hyperglycemia-induced overexpression of *cdc42* may be the reason for the polymerization of G-actin into F-actin, remodeling the cell structure, increasing the stiffness of the cytoskeleton and then impeding cell migration. There may be two reasons to elucidate this phenomenon. The competition for G-actin between different actin assembly factors has recently been demonstrated. Actin regulators compete with each other for a finite pool of G-actin to determine what type of actin structure is generated [45, 46]. The overexpression of *cdc42* may cause excessive polymerization of G-actin into F-actin and stress fibers so that the G-actin pool becomes deficient for generating other cell structures imperative for migration. The argument is supported by the finding in this study that the F-/G-actin ratio increased from 41% in normal glucose conditions to 52% in hyperglycemia. Otherwise, Rac1 and Cdc42 may compete and cooperate for finite G-actin to form different protrusive structures for cell migration [44]. Overexpressed and overactivated Cdc42 would occupy more G-actin to form filopodia, resulting in less lamellipodial formed by Rac1, and thus providing less traction force for cell motility. Another reason may be that overexpression of *cdc42* may cause G-actin to polymerase around the edge of a cell and then convert the cell from an anisotropic to an isotropic shape. In the present fluorescence image, we observed that the F-actin was located at the cell peripheral and arranged the cell phenotype as an isotropic, spread-out circle. Cdc42 is reported to be located at the tip of the leading edge and determines the polarity of migrating cells [47]. Cells with a spread-out morphology or larger spread area are known to have higher stiffness than cells with a small area or spindle morphology [39, 48]. However, the inhibition or global activation of Cdc42 results in disrupted directionality of migration through destructing the cell polarity [22, 49, 50]. Under hyperglycemic conditions, there is an overexpression and overactivation of Cdc42 in HUVECs, leading to the disruption of cell polarity and subsequent impairment in migration. As according to previous literature, Cdc42 can effectively regulate *WASP*, *RhoA* and *Rac1* genes, and actively participate in the actin cytoskeleton formation and cell movement [51–54]. Therefore, we can suggest that spatially localized rather than the overall activity of Cdc42 is important for the migration of HUVECs.

## Conclusion

In conclusion, this study indicates that hyperglycemia induces the overexpression of *cdc42* and overactivation of the downstream effector N-WASP/Arp2/3 signaling to promote the polymerization of G-actin into F-actin, which is located at the edge of the cell, resulting in increased stiffness of cytoskeleton, destruction of the polarity and reduction in the migration of HUVECs. This study employed the rate-jump method to measure the elastic modulus of adhesion cells accurately by AFM. The results imply that inhibiting Cdc42 in hyperglycemia may be a potential therapy for promoting tissue regeneration in diabetes, and so should help for various diabetic complications, e.g. the diabetic foot or stroke.

## Supplementary Material

rbae004_Supplementary_Data
