# Supplementary material for: Vascular endothelial cellular mechanics under hyperglycemia and its role in tissue regeneration
Source: Regen Biomater. 2024 Jan 25;11:rbae004. doi: 10.1093/rb/rbae004 (PMC10858347; doi:10.1093/rb/rbae004)
Supplement: rbae004_Supplementary_Data [file rbae004_supplementary_data.docx]

**SUPPLEMENTARY INFORMATION**

**Vascular endothelial cellular mechanics under hyperglycemia and its Role in tissue regeneration**

Kui Wang^1,2,#^, Yongmei Ge^1,#^, Yongshuai Yang^1,#^, Zhenjian Li^1^, Jiayi Liu^1^, Yizebang Xue^1^, Yuanjun Zhang^1^, Xiangchao Pang^3,4^, A.H.W. Ngan^2*^, Bin Tang^1,3*^

^1^ Department of Biomedical Engineering, Southern University of Science and Technology, Shenzhen, P.R. China

^2^ Department of Mechanical Engineering, University of Hong Kong, Pokfulam Road, Hong Kong, P.R. China

^3^ Guangdong Provincial Key Laboratory of Cell Microenvironment and Disease Research, Shenzhen Key Laboratory of Cell Microenvironment

^4^ College of Materials Science and Engineering, Central South University of Forestry and Technology, Changsha, P.R. China

^#^ The authors make the equal contribution for the article.

** Corresponding author, Email:* [hwngan@hku.hk](mailto:hwngan@hku.hk); [tangb@sustech.edu.cn](mailto:tangb@sustech.edu.cn).


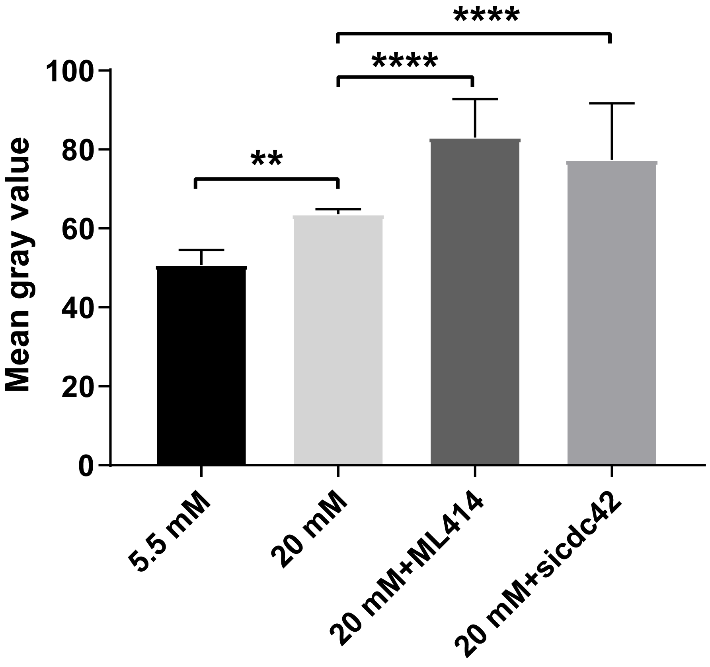


Figure S1 Mean gray values of F-actin cytoskeleton in HUVECs incubated for 24 hours under different glucose conditions. **p<0.01, ***p<0.001, ****p<0.0001.


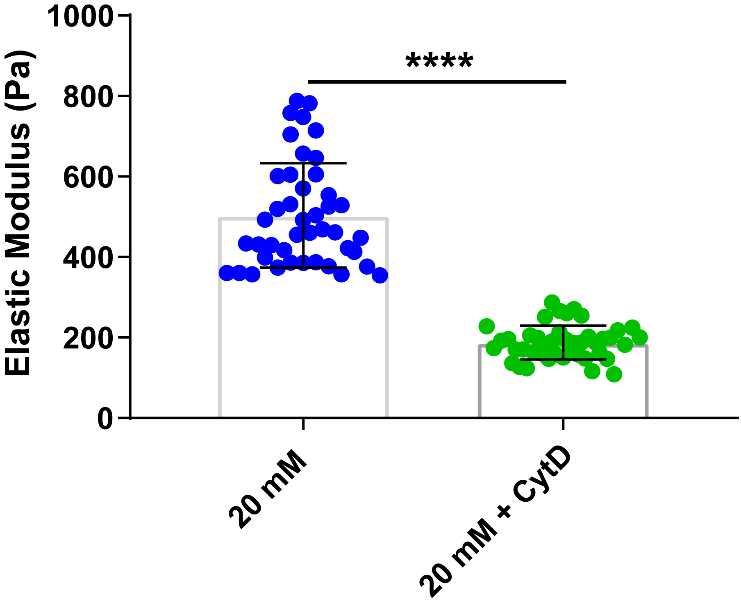


Figure S2 The elastic modulus of HUVECs treated with CyctD under 20 mM glucose condition. **p<0.01, ***p<0.001, ****p<0.0001.


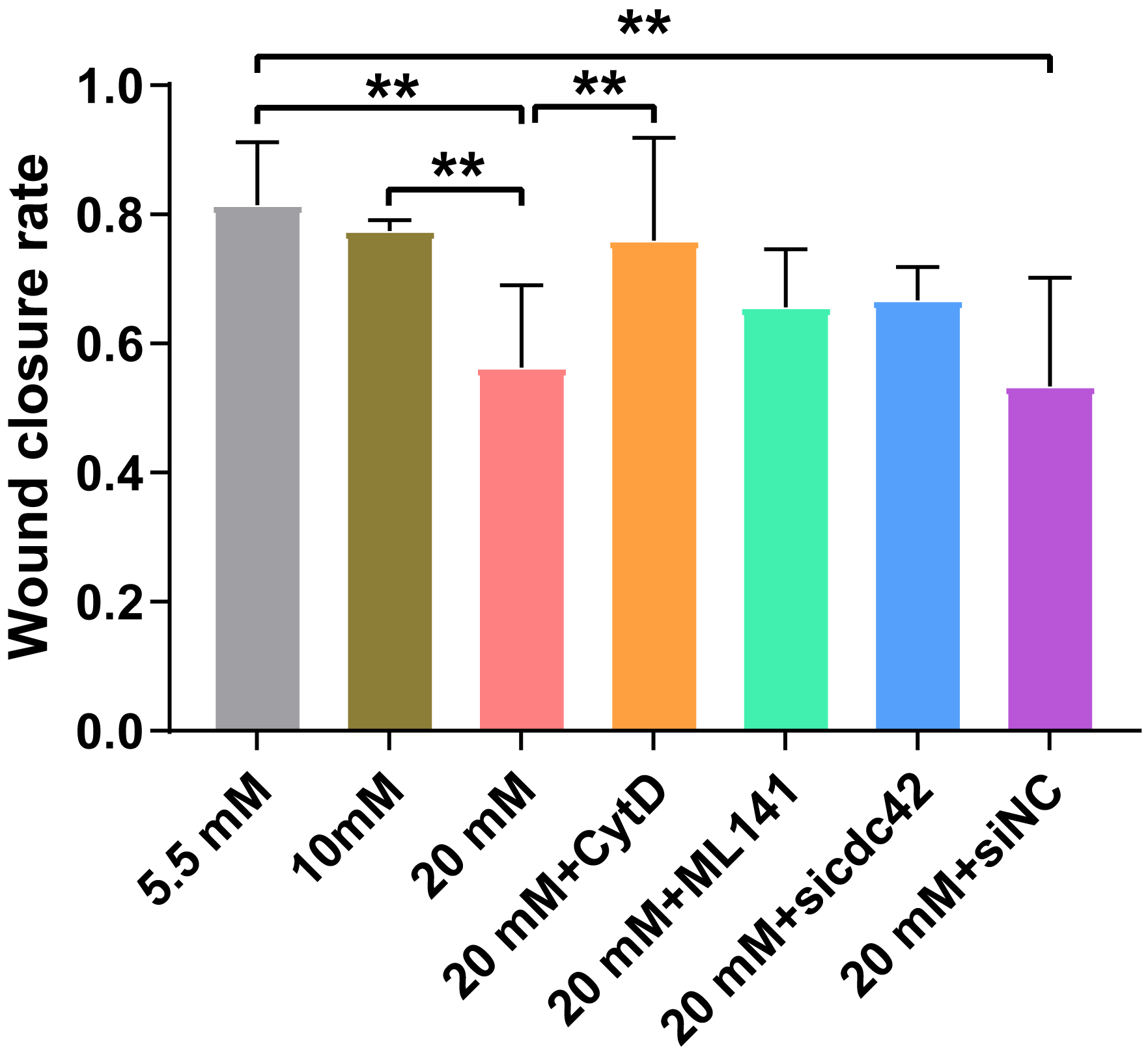


Figure S3 The wound closure rates of HUVECs under different glucose conditions. **p<0.01, ***p<0.001, ****p<0.0001.


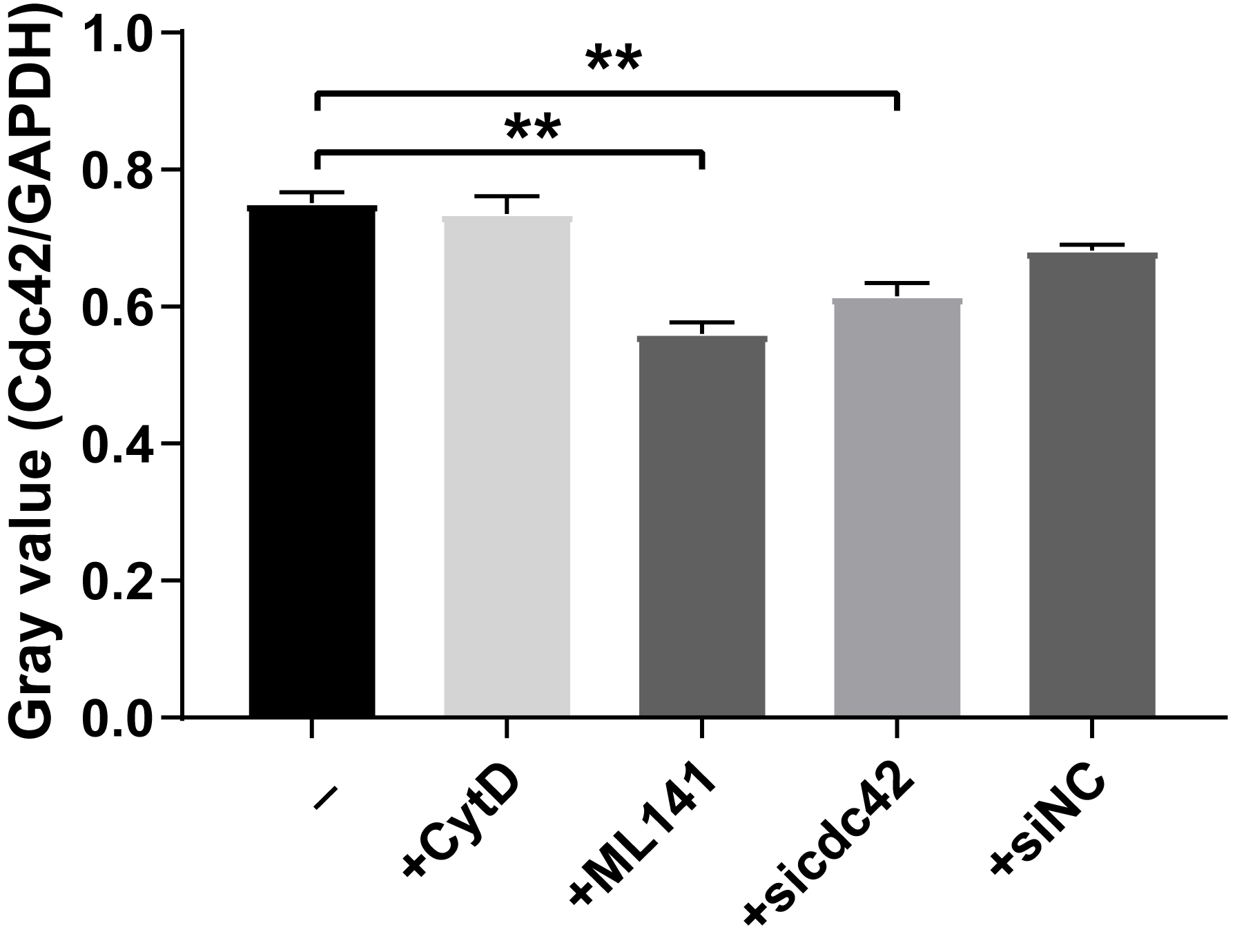


Figure S4 Gray values of Cdc42 expressed in CytD (1µM), ML141, sicdc42, and siNC under high glucose condition. **p<0.01, ***p<0.001, ****p<0.0001.
